# Medication adherence and economic problem among patients with type 1 diabetes in Central Java Province, Indonesia

**DOI:** 10.1186/1687-9856-2013-S1-P33

**Published:** 2013-10-03

**Authors:** Agustini Utari, Asri Purwanti, Rudy Susanto

**Affiliations:** 1 Division of Pediatric Endocrinology, Department of Pediatric, Faculty of Medicine, Diponegoro University, Semarang, Indonesia

## 

Diabetes type I prevalence is increasing around the world. The lack of knowledge, social economic problem, and the limitation of health systems infrastructure are the problematic issues in developing countries including Indonesia. The objective of this paper was to investigate medication adherence and economic problem among patients with type 1 Diabetes in Central Java Province, Indonesia.

This study is a qualitative study using in-depth interviews of 10 parents of children with type I Diabetes, recruited in Central Java Province, Indonesia. Broad interviews are used to provide information including parents’ experiences regarding their children’s treatment particularly medication-taking behavior, complementary and alternative medicine use and social economic problem in their families.

The parent’s biggest concern (70% of parents) is related with the high cost of longlife medication and laboratory evaluation. Some children have no insurance and eventhough some of them have insurance but not cover all the cost. Some barriers of medication adherence were related with economic problem and insulin injection which were scary for some young children. Complementary therapy including herbal treatment were usually used alongside of conventional treatment.

This study suggests that medication adherence were might related with economic condition in families with type I Diabetes in Central Java, Indonesia.

